# A Systematic Review of the Use of Blogs and Vlogs as Self-Expressors for People with Serious and Common Mental Illness

**DOI:** 10.1007/s11126-025-10158-2

**Published:** 2025-05-10

**Authors:** Iris María Muñoz-del-Pino, José Antonio Matías-García, Francisco Javier Saavedra-Macías

**Affiliations:** 1 Department of Experimental Psychology, Faculty of Psychology, University of Seville, Seville, Spain; 2 Hospital Universitario Nuestra Señora de Valme, Seville, Spain 41014

**Keywords:** Serious mental illness, Common mental illness, Blogs, Vlogs, Personal recovery

## Abstract

The use of blogs and vlogs by people with serious mental illness (SMI) and common mental illness (CMI) has attracted increasing academic interest due to the development of online illness narratives. This systematic review aims to explore the use of blogs and vlogs, identifying their narratives and characteristics, perceived benefits and adverse effects associated os their use. This would allow us to know the influence of this type of media on authors’ personal recovery. Following PRISMA guidelines, an exhaustive search of four databases was conducted, using terms such as ‘blog’, ‘vlog’ and ‘serious mental illness’ among the most relevant. The selected articles were empirical studies, written in English, published in peer-reviewed journals from 2014 to 2024 and explored the use of blogs and vlogs by people with SMI and CMI. Eighteen studies met the inclusion criteria. Self-expression narratives on these platforms tend to be first-person and address issues such as stigma, barriers to treatment and self-management of symptoms. Key benefits include the creation of support networks, reduction of social isolation and individual empowerment, all of which are intrinsically related to personal recovery. However, risks were also identified, such as the possibility of employment discrimination and emotional vulnerability to negative comments. This review highlights the need for future research that explores cross-cultural experiences and provides a sound basis for the design of interventions that promote the positive use of blogs and vlogs in mental health recovery.

**Supplementary Information:**

The online version contains supplementary material available at 10.1007/s11126-025-10158-2.

## Introduction

The beginnings of digital storytelling date back to the mid-1990s, although the “blog” feeling already existed previously [1]. Blogs and vlogs provide a medium for the expression of thoughts, feelings and opinions using Web 2.0. This can be in the form of cyber-blogging or through audiovisual content. One of the most prevalent typologies is the personal blog, in which individuals assume the role of storyteller, organising their publication in a chronological manner, akin to a traditional diary [2]. In a study conducted by Lenhart and Fox [3] it was found that most US bloggers had a primary objective in writing about their personal experiences, establishing social relationships with their readership, and sharing information. As indicated in the Digital 2024 Report [4] 19.7% of social network users engage in sharing aspects of their lives, 22.5% seek to meet new individuals, and 22.7% participate in the sharing and discussion of opinions.

The development of technology has also enabled the growth of the blog in various forms and platforms, including micro-blogs such as those on the Twitter application (now X) or Instagram. Additionally, there are video blogs, which are blogs broadcast on video in applications such as YouTube [4]. Similarly, patients have used both blogs and vlogs in the context of health-related discourse, particularly in the case of conditions such as cancer, HIV, arthritis and diabetes [5, 6]. Such spaces provide an environment in which individuals can openly express their emotions, exchange information and share social support with others [7–9]. Furthermore, reading and responding to other bloggers’ posts can assist in reducing distress, navigating uncertainty and influencing the identity reconstruction of those involved, as evidenced by Green [10] and Heilferty [11].

From a sociocultural perspective, the utilisation of communication technologies, such as the printing press in the 15th century or blogs and vlogs in the 21st century, is intrinsically intertwined with our psychological processes and the development of our subjectivity [12]. From the perspective of narrative theory, identity is understood as a personal story or narrative that is constructed to make sense of an individual’s own experience. This imposed pattern of meaning serves to make the experience meaningful [13]. Taylor [14] asserts that “having” a self implies understanding one’s own life narrative. In this case, the author positions himself as the protagonist of his life story within his own moral context. Consequently, narratives developed in online media render authors as “wounded storytellers” instead of disease bearers, diverging from the biomedical concept and focussing on one’s personal experiences pertaining to illness [15].

This narrative approach to identity has become a foundation for the recovery model in mental health. Accordingly, recovery is a central tenet in the development of interventions and strategies aimed at improving mental health outcomes for individuals with mental illness [16]. In terms of definition, a study by Law and Morrison [17] that sought to establish a consensus on the meaning of recovery in people with experiences of psychosis found that most participants thought of recovery as “achieving a personally acceptable quality of life” and “feeling better about oneself”. In this vein, there is a surge of recent studies exploring the relationship between recovery, quality of life and well-being [18–20]. A particularly noteworthy aspect of this topic is the development of the CHIME framework [21], which emphasises five key elements: connectedness, hope, identity, meaning and empowerment. The concept of connectedness encompasses the development of supportive relationships and the cultivation of a sense of community. Hope and optimism play a pivotal role in instilling a belief in recovery and motivating positive change. Identity, on the other hand, is concerned with the restoration of a positive self-concept and the overcoming of stigma. Meaning, in this context, is understood as a way of leading a purposeful life that is defined by personal goals and values and which integrates the experience of mental illness. Empowerment, finally, involves gaining control over one’s life, recognising personal strengths, and taking responsibility.

Despite this, the term recovery has undergone several modifications over time [22] as numerous investigations have attempted to clarify what categories underlie recovery [23, 24] and how the narratives of people with mental illness influence recovery [5]. Recovery narratives can be argued to have brought about a shift in narrative genres, shaping an entirely new and specific one [25]. However, Llewellyn-Beardsley et al. [5] argue that most of the recovery narratives studied so far are offline and that it would be interesting to examine them in online media, considering their diverse forms, structures and contents. If we understand recovery as a long journey or path [26], online recovery narratives would be a travelogue and those who participate as spectators in the blogging community would be the companions of the journey.

Heilferty [11] suggested that the study and analysis of blogs in which illness experiences are narrated could improve the individual-family-professional relationship and, at the same time, allow professionals to learn about the different meanings related to the illness process developed by bloggers. However, few studies have focused on exploring the use of blogs/vlogs as a means of expression among people with mental illness, their characteristics, benefits or harms [27]. Furthermore, most of the existing literature on this topic has been published in English-speaking countries, indicating bias and limiting the known evidence, so it would be interesting to include data from other sociocultural contexts [5].

To our knowledge, there is no current systematic review exploring blogs / videos as a means of expression for people with common and severe mental illness. However, a scoping review that explored the experiences of people with mental health problems through the use of blogs [28] highlights the need to align the concerns of health professionals in the public sphere with the benefits of sharing personal narratives related to mental health in the private sphere. Additionally, they report that there are few studies that explore blogging as a restorative experience or the benefits and detriments of blogging. Furthermore, they suggested that this form of practice should be incorporated into the treatment plan as an adjunct to more conventional therapeutic methods. However, it should be noted that there is currently no established methodology for the study of this type of media. Consequently, before this approach can be implemented, it is necessary to conduct rigorous research and analysis.

In view of the absence of systematic reviews, the potential advantages of blog interventions and the proliferation of methodologies in research, it is essential to conduct a comprehensive analysis and integration of the current blog/vlog literature. Addressing the following research questions will facilitate a more comprehensive understanding of the extent to which blogs/vlogs serve as a conduit for self-expression among these individuals and the perceived benefits and challenges associated with their use. This will enable us to better understand the potential of blogs and vlogs for the recovery of people with mental disorders.

RQ1: What features are present in blogs or vlogs created by individuals with severe mental illness or common mental illness?

RQ2: What are the perceived benefits of the use of these media by people with severe mental illness or common mental illness?

RQ3. What perceived adverse effects do these individuals associate with their use of these forms of media?

## Methods

### Search Strategy

The review was carried out according to the Preferred Reporting Items for Systematic Review and Meta-Analysis (PRISMA) [29]. A search was conducted in four databases (PubMed, PsycInfo, Scopus and WOS) for articles published in peer-reviewed journals with a publication date between March 2014 and March 2024. The search terms employed were: (blog* OR vlog* OR YouTube*) AND (“severe mental illness*” OR “severe mental disorder*” OR schizophrenia* OR “psychotic disorder*” OR “personality disorder*” OR depression* OR “bipolar disorder*” OR “anxiety disorder*”) in title and abstract.

### Inclusion and Exclusion Criteria

The studies included were of empirical nature, published in English in peer-reviewed journals and concerned individuals diagnosed with serious mental illness (schizophrenia, bipolar disorders, personality disorders) or common mental illness (depression, anxiety disorders) that included the use of blogs or vlogs. In accordance with the transdiagnostic definition of serious mental disorders (SMI) as a mental, behavioural or emotional disorder that has a considerable impact on an individual’s functional capacity and severely impairs the ability to perform one or more essential activities of daily living [30], we will consider these three specific disorders, although it should be acknowledged that there are other diagnoses or conditions under the broader category of SMI.

Consequently, articles that were not published in English in peer-reviewed journals were excluded. Furthermore, studies involving individuals who had not been diagnosed with a severe mental illness or common mental illness (depression, anxiety disorders), as well as interventions or studies aimed solely at caregivers, family members, friends or health professionals, were not included.

Following the elimination of duplicates, the titles and abstracts were subjected to review in accordance with the outlined criteria. This task was carried out by the principal researcher, Author 1, who reviewed 100% of the results. Subsequently, eligible articles were independently reviewed by Author 1 and Author 2.

### Data Extraction and Methodological Quality Assessment

In the course of data extraction, information was collected pertaining to the authors, the journal, and the objectives of the reviewed studies. To characterise their sample, we recorded whether the focus of the study was blogs or vlogs, the sampling method used, and whether the sample included individuals with SMI, CMI or both. For blogs and vlogs, variables such as the number of posts, words or minutes of video analysed were considered, as well as the number of comments, likes or visits if described. Similarly, the gender, age, diagnosis, socioeconomic level, employment status and country of origin of the creators of the content were examined. Regarding methodology, the type of study and data, materials and instruments and data analysis used were documented. The results data extraction involved the identification of relevant excerpts from the articles that were aligned with the objectives of this systematic review.

The methodological quality of the reviewed articles was evaluated by Author 1 and Author 2 using the Mixed-Methods Appraisal Tool (MMAT) version 2018 [31]. The MMAT is designed to assess quantitative, qualitative and mixed methods studies through two initial screening questions and five criteria that must be met (0–5 points). The quality of the studies was not evaluated to exclude them from further consideration; instead, the aim was to identify potential limitations of the included studies. Consequently, a detailed account of the criteria that were met and not met will be provided in the Results section, according to the recommendations set out by the authors of the MMAT.

Before starting to extract data from the entire sample and quality assessment, 28% of the articles were independently reviewed by two researchers, Author 1 and Author 2, to ensure reliability in these tasks. These articles were selected at random. The two researchers met and achieved consensus.

### Data Analysis and Data Reduction

A type of thematic analysis, known as template analysis [32] was employed for the analysis of the extracted results. This analysis commences with the formulation of an initial template, which is constructed a priori through the examination of existing literature or through the analysis of a preliminary data set. With the intention of guiding the analysis in its early stages, the study objectives were used as the initial template. Subsequently, the template was iteratively revised with the creation of new categories, the mixing of existing categories or the elimination of categories as necessary. This process was independently conducted by two researchers, Author 1 and Author 2. These researchers held routine meetings to share the results of their analysis and to agree on the categories thus far, achieving consensus on reliability. Following each meeting, the previously analysed articles were reviewed to implement the necessary modifications to the coding. This process continued until no more information could be extracted from the articles. The definite category system will be presented in the Results section.

## Results

### Study Selection

**Fig. 1:**
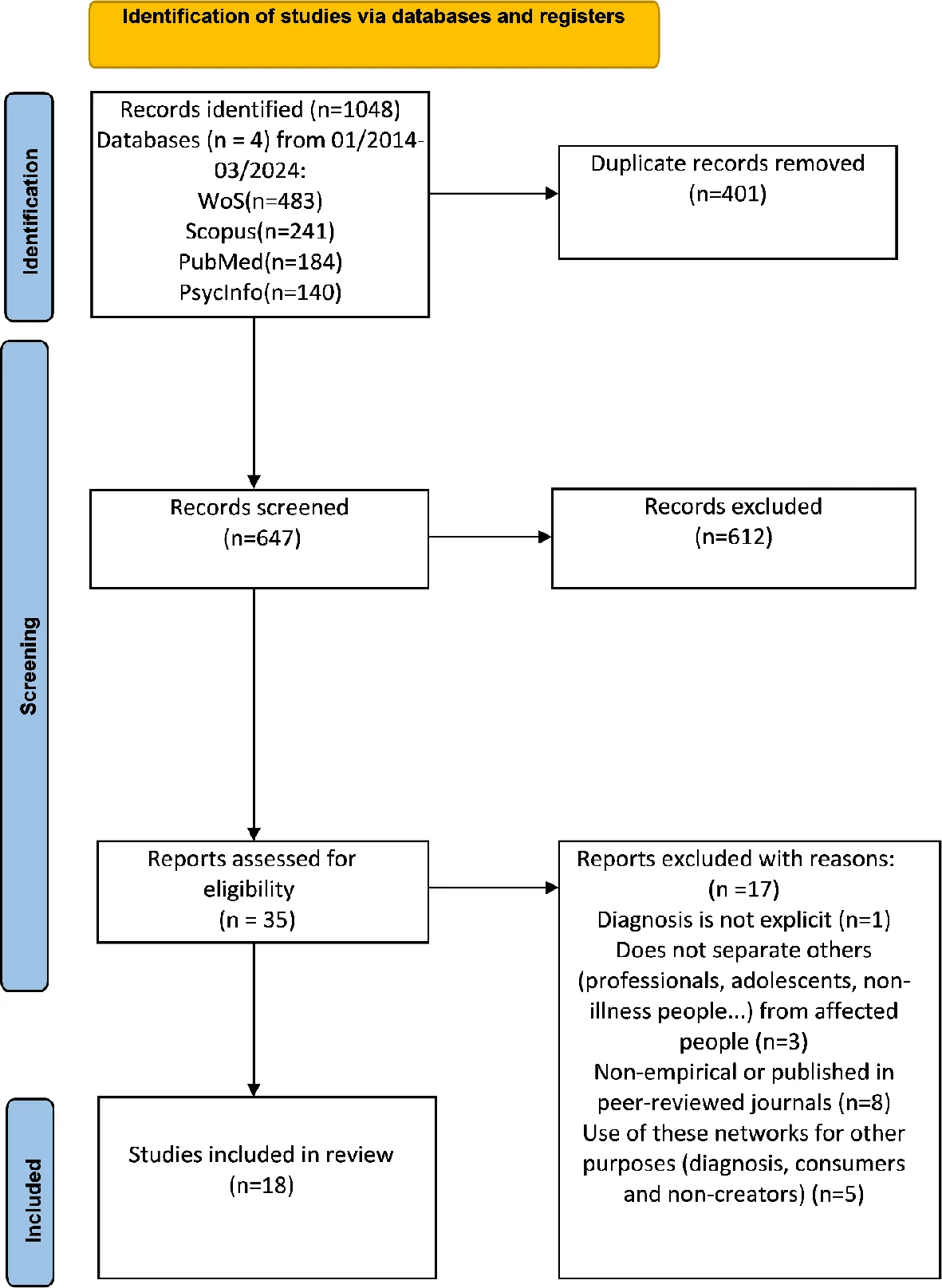
Study selection process flowchart

A total of 18 articles met the predetermined inclusion criteria and were therefore included in the systematic review. This process is illustrated in Fig. 1.

### Characteristics of the Articles

The following section will provide an overview of the most relevant characteristics of the studies reviewed. For a comprehensive understanding of each study, we refer to the detailed descriptions in the Electronic Supplementary Material.

The goals of the reviewed articles mostly focused on the analysis of how different mental illnesses were portrayed in blog/vlogs (narratives, themes, metaphors, words, etc.). (*n* = 11). The goals were related to narratives about treatment and interaction with medical professionals (*n* = 3), the interaction with consumers, media or other agents (*n* = 2) and the impact of blogs/vlogs on content creators (*n* = 2). Similarly, 13 articles examined blogs, while 5 investigated vlogs.

Regarding the sample, 6 articles included individuals with SMI, 8 included individuals with CMI and 4 articles included both. Most of studies employed individuals who self-identified as having the disorder(s) of interest (*n* = 17). Only one study selected participants with SMI diagnosis explicitly recognised by an external agency (*n* = 1). No studies performed a researcher assessment to confirm the diagnosis or severity analysis. The diagnoses represented in the articles were bipolar disorder (*n* = 7), schizophrenia spectrum (*n* = 5), borderline personality disorder (*n* = 1), depression (*n* = 11), obsessive compulsive disorder (*n* = 2), and generalised anxiety disorder (*n* = 1). One article did not record the specific diagnoses. Three included only women authors, five both women and men and 10 articles did not register the gender of authors. Only four articles recorded the age of bloggers/vloggers. One had 18 to 30 years old authors, one had 35 to over 50 years old and two had a diverse age range, from 18 to over 50 years old. Fourteen articles did not record the age of the creators. Only one article included a diverse socioeconomic background of the authors, while the rest (*n* = 17) did not record the socioeconomic backgrounds. The employment status was recorded in only two studies. The represented countries were the USA (*n* = 4), Spain (*n* = 3), UK, Canada, China, and Finland (*n* = 1, each). Nine articles did not record the country of the origin of authors.

Regarding sampling strategies, the studies employed a variety of methods, including intentional sampling (*n* = 8), a systematic search on platforms of interest (*n* = 4), simple random sampling (*n* = 1) or a combination of sampling methods (intentional sampling, systematic search, simple random sampling and/or virtual snowballing; *n* = 5).

Most of studies were qualitative (*n* = 12), while the remaining were mixed (*n* = 5) and quantitative studies (*n* = 1). Qualitative studies primarily employed thematic analysis (*n* = 8), interpretative phenomenological analysis (*n* = 1), computer-mediated discourse analysis (*n* = 1), metaphor analysis (*n* = 1) and descriptive analysis (*n* = 1). The mixed studies used a combination of thematic analysis (*n* = 2), metaphor analysis (*n* = 2), scalded reading (*n* = 1) with ANOVA (*n* = 2), correlations (*n* = 1), Chi-square test (*n* = 1) or term frequency-inverse document frequency (TF-IDF) (*n* = 1). The quantitative study used a variety of analytical techniques, including frequency counts of word categories, ANOVA, correlations, and principal component analysis.

Methodological quality analysis using the MMAT tool showed that the articles scored between 3 and 5 (*M*: 4.39; *SD*: 1.10), except for one article that scored 1. Regarding the weaknesses found in the qualitative articles, in two of them the data analysis procedure was not explained, and the internal consistency was insufficient [33, 34]; meanwhile, in another two, more data is needed to establish their [34, 35]. In the mixed articles, Van de Ven and Van-Nuenen [36] did not conduct a systematic qualitative analysis of the data but rather selected a few topics and data of interest as a sample of different methodological procedures, making it difficult to draw conclusions. In Woloshyn and Savage [37], the sociodemographic characteristics of bloggers were not described, although describing bloggers was one of their objectives. As for the only quantitative article, Fineberg et al. [38], the blog sampling strategy and data extraction do not allow for a representative sample of the blog population.

### Synthesis of Results

#### Features of Blogs/Vlogs of CMI and SMI Experiences

##### Narrative Themes and Characteristics

In our systematic review, there were eleven articles that analysed narrative themes and characteristics. Six included a SMI sample, while five articles included a CMI sample. Both the SMI and CMI articles seem to present mostly similar themes and characteristics.

First, one of the topics that bloggers/vloggers discuss in their content is the reason why they have a blog/vlog. As outlined by Sangeorzan et al. [35] and Woloshyn and Savage [37], one of the authors’ main objectives in creating a blog/vlog was to educate the public on mental health issues, provide positive role models and combat the stigma surrounding mental illness. By revealing their mental disorder in a public forum, they sought to normalise their situation within the broader social context. The content they created was designed to help other people and foster a sense of community, thus reducing both their own and consumers’ social isolation and consumers’ self-stigma, reconceptualising ‘normality’ [35]. Similarly, another reason was for blogs/vlogs to serve as a form of self-therapy [35, 37].

Most blogs / vlogs include narrations of personal illness experiences [36, 39, 40], generally employing a first-person discourse [37, 40]. Johnson et al. [41] also discussed the use of third person in personal experience narratives in Black Americans’ vlogs to depersonalise narratives and avoid difficult situations and uncomfortable feelings. Through their personal narratives and informative content, the bloggers/vloggers discussed clinical aspects such as the causes of mental illnesses, diagnoses, interactions with medical and mental health professionals, medical and/or psychological treatments, and side effects and their management [42–44]. In addition, the social aspects related to people with SMI and CMI are also described. These include social activism initiatives, stigma experiences and barriers to treatment [35, 37, 41]. Some of these barriers include personal histories of trauma, rejection of formal mental health systems, invisibility of mental disorders or experiences of racism [41].

Regarding how narratives are constructed, Conneely et al. [45] observed the coexistence of biomedical and moral understandings of mental illness in online depression blogs. First, the biomedical view of depression described the condition as a brain-based disorder comparable to other medical conditions. The diagnosis and medication offered legitimacy to the problems of the bloggers, providing a separation between self-perception and depressed behaviour, and deflecting blame and shame. This view was often presented to contest and oppose a moral view of depression, in which depression is regarded as a meaningful reaction to social circumstances and the individual is seen as having agency. However, the same bloggers later described their recovery in moral terms. Recovery was presented as a deliberate transformation in how people perceive the world and live their lives, being in control of their mental health. These two views could also be intermingled in recovery, with the moral view serving to counteract biological processes.

The only article to provide a classification of narratives is that by Xu and Li [46], which analysed narratives of depression on the Chinese platform Douban. In their study, the researchers identified four different types of narrative. Complaining narratives represented the authors’ reflection on external causes of their depression, including family control or neglect, bullying, stress, noncompliance with dominant social norms and homophobia. The regret narratives included narrations of internal factors of depression, including personality traits, perfectionism, lack of self-disclosure and/or the repression of emotions (compliant behaviours). Superiority narratives were related to the expression of being smarter and having a higher moral sense than others. These narratives disregard biomedical explanations and attribute their depression to a moral outcome. Finally, discovery narratives reflected a novel understanding of self-self, others and key events, reframing their narratives of depression and expressing positive emotional and interpersonal changes in the present.

Lastly, in her analysis of the most successful bipolar disorder bloggers, Egher [42] expanded the concept that Collins and Evans [47] termed ‘interactional expertise’. Egher [42] defends that bloggers can become expert online mediators between the professional community and consumers. These bloggers demonstrate linguistic prowess in medical knowledge of bipolar disorder, explaining medical phenomena in a more accessible language, giving advice about treatments and criticizing certain habits of practitioners. They maintain a position of intermediary, often positioning themselves as complements or alternatives to medical professionals. They draw on experiential and scientific sources of knowledge, making strategic decisions regarding the construction of their discourse. These influential bloggers also engage in collaborative efforts with professionals and researchers, which serves to reinforce their image of expertise. Lastly, they expand their mediation work by developing close relations with mass media outlets (documentaries, radio programmes, talk shows, books or conferences), thereby further increasing their influential standing.

##### Use of Language and Literary Devices

A total of nine articles included in the review discuss the use of linguistic elements and literary devices present in distinct blog or vlog content. The remaining articles address individuals with SMI (*n* = 4), individuals with CMI (*n* = 4) or individuals with SMI or CMI (*n* = 1).

Regarding linguistic considerations, first-person pronouns and negative emotions were used with greater frequency in blogs written by individuals diagnosed with psychosis compared to blogs written by individuals with other illnesses, CMI or non-illness groups [38].

The usage of words associated with biological processes did not differ significantly between blogs created by individuals diagnosed with psychosis and those written by individuals with other illnesses or CMI [38]. The use of the word "stigma" or similar terms was identified likewise in studies of individuals with SMI (*n* = 2) [48, 49] and CMI (*n* = 2) [36, 50]. In addition, the use of emoticons to express empathy or virtual actions that translate into tangible actions, such as a virtual hug, is also observed in depression blogs [39].

In terms of literary devices, metaphors were the most frequently referenced (n = 8), followed by comparisons or ellipsis (n = 2). In relation to the metaphors used in studies of individuals with SMI (n = 4) [40, 43, 48, 49], one of the studies indicated that there is a greater use of metaphors in blogs authored by individuals with SMI compared to blogs authored by health professionals [48]. However, no significant differences were observed between patients according to diagnosis, which further support to the “severe mental disorder’.

Regarding the subject matter of metaphors, four main themes are identified: war and journey; "split-self"; disorder as a living entity; and disorder as darkness, descent or container metaphors.

The metaphors used the most frequently by SMI bloggers were those related to war/struggle and journey (*n* = 4). In contrast, in articles about people with CMI (*n* = 3) [36, 39, 50], most of the metaphors that appeared are more related to struggle or battle. Only Van De Ven and Van Nuenen [36] referred to the utilisation of journey metaphors in individuals with depression, however, in this instance, depression was regarded as a key factor in the process, playing a role akin to that of a partner on the journey.

In the context of war metaphors, the disorder and the factors associated with it, such as social stigma, prejudice, medical treatment, symptoms (including negative emotions), society, and even patients themselves are regarded as enemies (*n* = 6) [36, 39, 43, 48–50]. The use of war metaphors can serve to empower the author when he confronts his enemies or, conversely, can contribute to a bleak and dispiriting tone when the author is reduced to the battlefield [48, 50]. On the contrary, the experience of living with the disorder was often conceptualised as a journey (*n* = 3) [40, 48, 49], with the objective of attaining emotional well-being or acceptable living conditions. It should be noted that the domains that were represented as adversaries in war metaphors were also present in journey metaphors as obstacles or locations of transit [49, 50]. However, there are exceptions to this, with stigma manifesting as a burden and disorder potentially functioning as a companion [49].

The concept of the “split-self” was also present in blogs of individuals with SMI (*n* = 3) or CMI (*n* = 1) [50]. In war metaphors, the “other self” was conceptualised as an adversary (*n* = 3), signifying an internal schism and a conflictual relationship with the self [43, 48, 49]. However, in journey metaphors, the self remained intact, without a separation between the ill self and the healthy self [49]. Other types of metaphors referring to depression appear in the articles reviewed, but to a lesser extent, such as depression as a force, a thing or a weight [48].

Next, authors who have explained the use of the metaphor of the disorder as a living entity by people with SMI (n = 2) [43, 48] or CMI (n = 3) [36, 39, 50] have found that it can be perceived both in positive and negative ways. On a positive note, some patients described the disorder as a "being that could be lived with”, indicating a sense of co-existence and acceptance [49]. One example of this is that one blogger connected the ‘ghost’ in a song to their own personal metaphor from their younger years, the "corner man", and this figure personified their depression as a partner [36]. Conversely, the disorder is also depicted as a malevolent entity, monster or beast, evoking fear and a constant struggle against it [39, 43, 48, 50]. This fear is likely to intensify when the individual in question has internalised the monster or disorder, identifying with it or personifying it as a part of themselves [43].

Finally, the metaphors of disorders as darkness, descent and container will be discussed, which are related. The use of darkness as a metaphor is associated with feelings of hopelessness and entrapment in individuals with SMI [48] or CMI [39, 50]. These metaphors portrayed the disorder as being located within an enclosed and dark space, like "being in a well” [48]. Such imagery evokes a profound sense of hopelessness and entrapment. Furthermore, the concept of darkness was frequently personified [39], thereby reinforcing the predominantly negative perception of the disorder [48]. According to this line of argument, the ideas of descent and container are employed to elucidate the oppressive quality of mental illness. In their accounts, bloggers described a process of "falling into the state of illness" [48] which is linked to a sense of being in a dark and enclosed space [50]. In this context, the use of balance metaphors emphasises the notion of a loss of emotional control and stability experienced by bloggers [50], employing depression as a metaphor for an uncontrollable descent into darkness or as a destabilising force affecting emotional equilibrium.

In addition to the use of metaphorical language, other literary devices were employed as comparisons (*n* = 1) or ellipsis (*n* = 1). Xu and Li [46] described the use of positive comparisons (*n* = 1) by people with depression in relation to superior intelligence and high morality. Phrases such as “being like angels” or “God-given intelligence” illustrated this tendency to see positive aspects in traits associated with depression. The use of ellipsis (*n* = 1) was also found, as we can see in the phrase “empty hole”, which emphasised the feeling of emptiness in people with depression [39].

##### Clinical Issues: Symptomatology, Medical Treatments and Therapies

8 of the 9 articles related to people with SMI [33, 35, 37, 38, 40, 43, 48, 49] and all articles related to people with CMI (*n* = 8), focus on clinical aspects. Additionally, this reference can be found in the article concerning people with SMI and CMI (*n* = 1).

In terms of symptomatology, the clinical manifestations of bipolar disorder encompassed a range of symptoms, including anxiety, depression, anger, hallucinations, alcohol and drug-related problems, suicidal behaviour, erratic behaviour, interpersonal difficulties, work-related challenges and insomnia [43]. The experience of mania was described in a variety of ways. Some individuals reported an increase in energy, which they perceived as a positive experience. However, others described it as a consistent behavioural pattern that led to interpersonal tensions and other difficulties, including rapid speech, racing thoughts, delusions of grandeur and inflated self-esteem [43]. In borderline personality disorder, the symptoms cited were found to align with the diagnostic criteria outlined in the Diagnostic and Statistical Manual of Mental Disorders, Fifth Edition (DSM-V). The most prevalent symptoms were unstable relationships, fear of abandonment or self-harm, followed by affective instability, suicidal ideation / behaviour and intense anger, among others [37].

Individuals with depression exhibited symptoms of isolation, seclusion, emotional suffering, overwhelming desires to sleep or suicidal thoughts [36, 39, 46]. Additionally, long-term undiagnosed depression was associated with blame, isolation and suicidal thoughts, highlighting the severe impact of untreated mental health conditions [41]. Furthermore, the symptoms of anxiety disorders frequently involved worry or panic, social anxiety and panic attacks, often exacerbated by trauma or genetic factors [51].

Regarding medical treatments, specific medications such as olanzapine [49], cymbalta, amantadine and phentermine have been identified in blogs as having an impact on symptoms, including tremors and tics [33]. However, there was a mixed perception regarding the efficacy of generic medications compared to brand-name [33]. Opinions on pharmacological treatments were found to vary considerably, with some patients reporting that they found them essential and effective, while others reported experiencing negative side effects or feeling that they were ineffective [37, 43]. Among vloggers, participation in treatment was commonplace, encompassing a range of modalities, including medication and hospitalisation [37]. Furthermore, bloggers expressed frustration with the mental health care they received, mentioning issues such as medication, misdiagnosis and institutionalisation [38, 40] in the case of people with SMI and self-medication, prescription [36] and the restrictive capacity of health services [50] in the case of individuals with CMI. However, there were also some positive views about psychiatrists and the importance of having a good relationship with mental health providers [40].

In relation to therapies, it became evident that a holistic and personalized approach to mental health care is of great importance in both the context of severe [49] and common mental illness [44]. The efficacy of the treatment was contingent on the timing and flexibility of the mental health services provided, with interventions being made available at a time when the patients were ready and able to participate [44]. On the plus side, some people shared their positive experiences and encouraged others to seek help. It was also noted that it is important to find a suitable therapist “that looks like you”; this was a crucial consideration in the context of African-American bloggers with depression [41].

There were few studies that discussed experiences in blogs / videos with therapies for people with SMI, except for Woloshyn and Savage [37] that mentioned interventions such as mindfulness, counselling, and dialectical behaviour therapy with positive results for individuals.

With regard to CMI, cognitive-behavioural therapy (CBT), self-help and support groups have been identified as efficacious treatments for anxiety, with specific techniques, such as breathing exercises for the management of panic attacks [51]. In the context of depression, Johnson et al. [41] also cited the utilisation of support groups as a possible intervention.

It was imperative to adopt a holistic approach to rehabilitation that considered the influence of personal, interpersonal and environmental factors [44]. This involved the provision of comprehensive support that extended far beyond the scope of mental health services.

#### Perceived Benefits from Sharing Experiences of CMI and SMI in Blogs/Vlogs

In our systematic review, there were thirteen articles that studied perceived benefits from sharing mental health experiences. Eight included a SMI sample, while five articles included a CMI sample. The benefits described in the articles are consistent in both SMI and CMI.

First, authors use blogs as a therapeutic tool [35, 37, 41]. The act of creating blog/vlog content, engaging in self-reflection, facilitated a deeper understanding of themselves [37]. Other bloggers indicated that the process of writing posts served as a coping mechanism, enabling them to navigate the challenging circumstances of their lives [40]. Similarly, the authors discussed the influence of positive feedback, leading to an improvement in their well-being and self-efficacy [35].

The practice of blogging/vlogging facilitates the reduction of social isolation and the creation of a sense of community [35, 39, 40, 46, 48]. The use of the Internet enables individuals with mental disorders to establish connections with others who have experienced similar circumstances. This facilitates the reconceptualization of “normalcy”, fighting against public and self-stigma [35, 37, 39, 41]. The online network serves to foster social solidarity and provide protection from negative online comments [40]. Furthermore, it enables the dissemination of information about mental health advocacy initiatives [37, 41]. Online communities facilitate the breakdown of communicative barriers frequently encountered by individuals with mental disorders, attributable to both their disorder and the challenging social circumstances in which they find themselves [35]. Anonymity lessens the pressure to conform to socially accepted positive narratives of “restitution”; online spaces become a safe and more intimate space in which to discuss problems freely, sharing thoughts, struggles, hopes and fears [36]. In fact, it frequently serves as a platform for people with mental health conditions to express their frustrations with mental health medical professionals, including those related to medication, incorrect diagnoses or the risk of institutionalisation [40]. In blogs [44], the importance of allowing time and space for reflection on one’s situation was mentioned in order to take steps for recovery. Online narratives serve as a source of inspiration for individuals to challenge the barriers of silence and access to treatment, empowering them to create their own blogs [37] or to seek professional help for the first time [41].

Additionally, online spaces become places for social learning and consultation [33]. Blogs/vlogs facilitate the development of patient knowledge [33], becoming spaces for exchange and reciprocity [40]. In their blogs and vlogs, individuals provided practical advice based on their experiences for interacting with healthcare professionals, medication use, seeking mental health support, learning evidence-based treatments, stress awareness, emotional regulation and coping mechanisms, meditation, music, healthy sleep or nutrition routines and relationships with loved ones [37, 40–44]. The sole article that made use of blogs as an intervention technique revealed greater and better usage of computers amongst its beneficiaries [34]. It is important to note that the design and structure of blogs facilitate greater access and concentration of information than is possible with analogue media [33]. The number of comments, likes, and other features of blogs/vlogs enable users to identify the most relevant and popular content with ease.

Lastly, especially influential bloggers/vloggers can also have an impact on research and social-political debate [42]. When working with researchers, they can assist in the enrolment of a substantial number of study participants and provide experiential knowledge and valuable insights into pertinent areas for future research, as is the case with collaboration with patient organisations. Additionally, they can utilise their online expertise for more political purposes, as they may generate public sympathy, emphasise the urgency of specific legislative or treatment provisions.

#### Perceived Adverse Effects from Sharing Experiences of CMI and SMI in Blogs/Vlogs

In the course of our review, we identified four articles pertaining to individuals with SMI and one article pertaining to individuals with CMI that examined the perceived adverse effects of sharing their mental health experiences on blogs and vlogs. The authors identified several key areas for attention, including the perceived technical challenges associated with the adoption of this novel technology [34]; concerns about job seeking due to stigma after disclosing their mental illness [35]; sensitivity to feedback and the impact of negative comments [37, 39, 40] and ethical implications of using these media [39]. In this sense, some blogs / videos could foster negative symptomatology for consumers through certain publications.

#### Platform and Blog/Vlog Format Characteristics

Some articles analysed different platforms and blogs/vlogs with different characteristics (*n* = 3); one studied SMI platforms and content and two studied CMI.

The features and target audience of blogs can vary depending on the platform, which in turn affects the type of user discourse and interaction. Van de Ven and Van-Nuenen [36] examined various platforms from this perspective. Platforms such as SANE and Time-to-Change (TTC) are more text-based, with few hyperlinks, are moderated and edited prior to publication and user interaction is low. In contrast, Reddit allows posts to be immediately posted and rated, acquiring better positions in the feed and establishing a meritocratic structure. In Reddit, moderation is conducted retrospectively and is guided by a set of established rules, such as prohibitions to diagnose others or advocate for or against specific treatments. Furthermore, the degree of anonymity varies between platforms. In addition, Van de Ven and Van-Nuenen [36] studied how different types of narrative are constructed within each platform, reflecting different blog cultures: Reddit includes mainly monologues and have a greater usage of emotional terms, profanities, SSRI commercial names and the word “weed”, while TTC acquires a more narrative perspective and SANE is more focused on creativity.

**Fig. 2:**
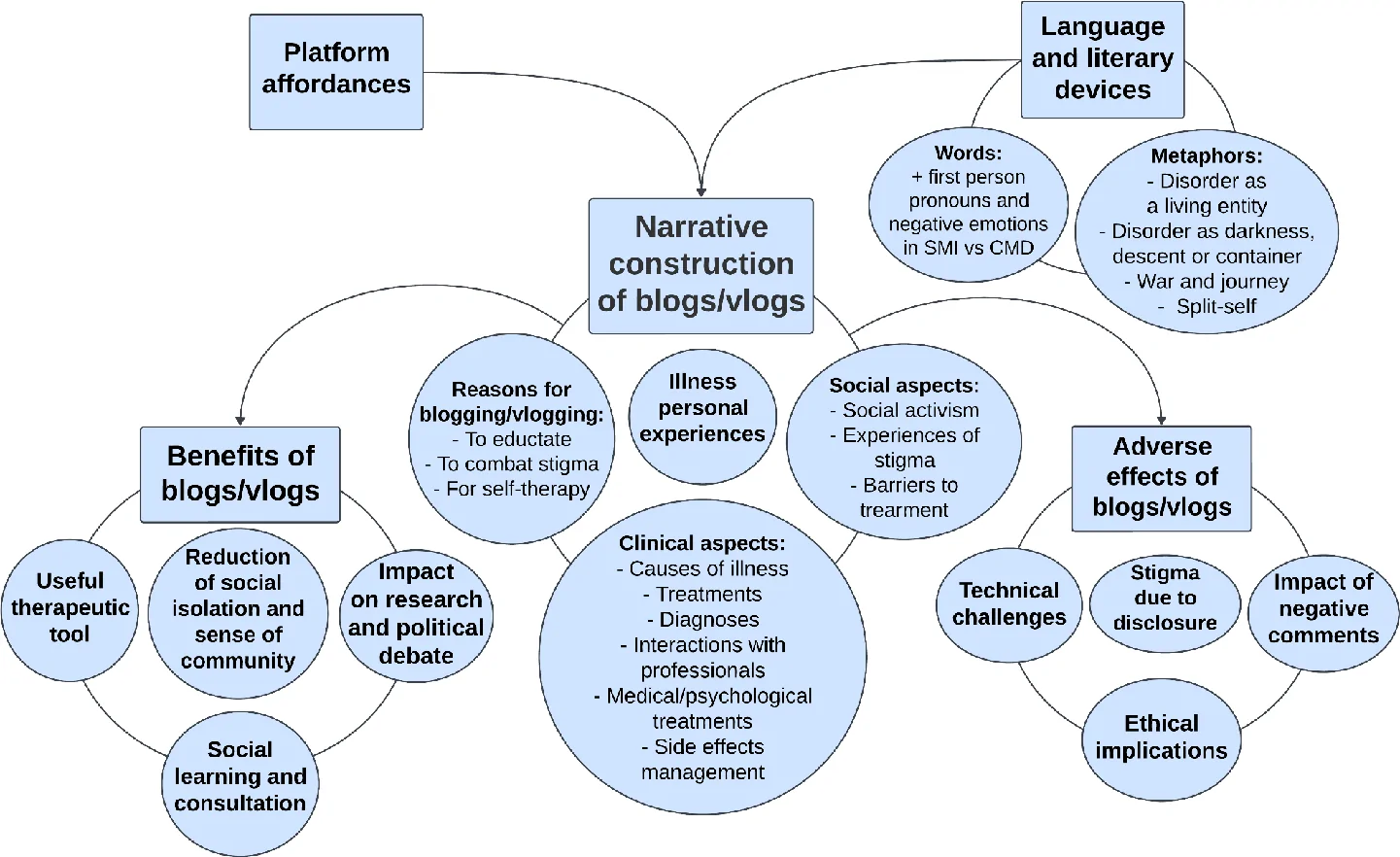
Theoretical model that integrates the systematic review main findings

Vlogs can also have different characteristics. Today, there is an increased tendency to upload vlogs that are monologues [37] and consumer-generated, as opposed to professional-generated content [51]. The number of views for consumer-generated and professional-generated vlogs is equivalent [51]. However, mixed media presentations, which include music, images, text, notecards, or voice-overs, tend to receive more views, are shorter in length and exhibit a more negative orientation towards treatment than monologues, which are more positively orientated [37]. A model that integrates the systematic review main findings is presented in Fig. 2.

## Discussion

To our knowledge, this constitutes the first systematic review to explore the use of blogs and vlogs as a channel for self-expression among people with SMI and CMI, with a view to identifying the features they offer and the perceived benefits and adverse effects associated with their usage. This will allow us to better understand the potential of blogs/vlogs for the recovery of people with mental disorders.

To be specific, although most of the reviewed articles did not employ the concept of recovery in their analysis, our results show many significant relationships with mental health recovery models. To provide a more comprehensive discussion, the CHIME recovery model (Connectedness, Hope, Identity, Meaning, and Empowerment) will be used as a framework for interpreting the features, benefits, and adverse effects of the use of blogs/vlogs by people with SMI and CMI.

Firstly, regarding the concept of Connectedness, it is important to acknowledge the significant role that peer support plays in facilitating the sharing of experiences and fostering mutual understanding within the context of social networks [52, 53]. In this line, De Silva et al. [54] highlighted the impact of social capital on mental health, emphasising the importance of community and social support. This social capital may decrease due to self-stigma [55], which reduces trust and reciprocity within communities and creates barriers to help seeking, exacerbating their isolation. In this context, blogs and vlogs created by individuals with SMI and CMI share online content to support their peers and foster a sense of community, thus reducing social isolation and self-stigma and reconceptualising ‘normalcy’ [35, 39, 40, 46, 48]. The anonymous nature of these spaces, therefore, reduces the pressure to conform to social acceptability and provides a safe environment in which to talk freely about problems, opinions and thoughts [36, 40].

Hope is another crucial concept in this context. It is of the utmost importance that people experiencing mental health challenges have hope and the opportunity to believe in the possibility of recovery. This belief is reinforced by the motivation for change, which influences the capacity of individuals to make significant changes in their lives [21]. Furthermore, creators of SMI and CMI content not only combat the stigma associated with mental illness, but also serve to motivate both themselves and their followers to have the power to control and modify their own lives [35, 37] which is also related to empowerment. In this line, having a sense of possibility is an effective method of maintaining an optimistic outlook on the future [56]. This is achieved by bloggers using positive metaphors or discovery narratives to reframe the experience of mental illness in a more constructive manner. For example, depression can be depicted as a companion or associated with positive qualities [46, 49]. Furthermore, the pursuit of dreams and aspirations provides direction and purpose [56]; at this juncture, bloggers’ narratives of personal and professional success inspire others to seek treatment and pursue their goals [40, 41].

Moreover, the reconstruction of a positive Identity enables individuals to perceive themselves beyond the constraints of their mental illness [57]. The act of sharing experiences on blogs and vlogs enables individuals to redefine their Identity and gain a deeper understanding of themselves, thus reducing the stigma associated with mental health [35, 37]. Some of Lakoff and Johson’s [58] statements can help us to account for the proliferation of the use of metaphors by bloggers and vloggers. These authors in their systematic analysis of metaphors claim that these shape human thought processes, as we relate to the world through metaphors. In this way, metaphors that are outside our conventional system can give new meaning to our experience and an alternative coherent structure to it. The use of metaphors, such as the "divided self", in blogs/vlogs may reflect how individuals perceive their identity as fragmented or in conflict due to illness [43, 48]. It also serves to demystify the discourse surrounding mental health, thus reducing the stigma associated with it [59]. Additionally, although some creators perceived the stigma in the workplace as a detriment, they nevertheless decided to present their authentic selves in an effort to challenge this stigma [35]. In this line, it should be considered that the receipt of negative feedback can have deleterious effects, such as the challenge of Identity and the fostering of feelings of stigmatisation [39, 40].

Otherwise, the concept of Empowerment is also noteworthy. This is related to the concept of expertise, as proposed by Egher [42], where mental health bloggers mediate between professionals and their peers. Using their experience and knowledge to explain medical phenomena accessible and critically, they not only reinforce their own sense of competence and authority but empower their followers by providing valuable and relevant information. This assertion is further substantiated by the findings of a study conducted by Monks-Woods et al. [52], which revealed that consumers of vlogs created by individuals with bipolar disorder reported that engaging with such content had a beneficial impact on their recovery process, leading to improvements in their overall well-being, coping strategies and offline confidence. Empowerment of individuals facilitates the ability to make well-informed decisions regarding their treatment plan and the therapeutic measures they undertake [33, 37, 43].

In summary, it can be posited that the use of online platforms, such as blogs and vlogs, can facilitate the recovery of people struggling with SMI and CMI. These platforms provide a conduit for social interaction and the development of positive relationships, instilling a sense of hope in the recovery process, enabling the formation of a positive identity and thus fostering empowerment.

Because of the aforementioned points, the empowerment achieved through these platforms enables individuals to construct critiques of health services, which has been discussed at length in numerous blogs and vlogs. A common thread among these accounts is the frustration expressed about misdiagnosis of medication, institutionalisation and the perceived restrictive capacity of health services [36, 38, 40, 50]. It is also important to note that institutions lack the flexibility and personalisation required for effective treatment [44]. Furthermore, the relationship with mental health providers was found to be inadequate and negative experiences with psychiatrists were also recurring themes [40]. As Saavedra et al. [19] have observed, patient dissatisfaction with mental health services is often associated with the perception of impersonal and structured care that does not adequately address their individual needs. Furthermore, studies such as that conducted by Perkins and Repper [60] have highlighted persistent stigmatisation within the health system, which has an adverse impact on the quality of treatment received by patients with severe mental illness.

Considering the discussion and the results obtained, it can be posited that there are notable parallels between the narratives presented by individuals within the SMI and CMI subcategories, as well as their exhibited characteristics within their respective blogs or vlogs. Furthermore, Coll-Florit et al. [48] established that there were no significant differences between patients according to diagnosis, which reinforces the category of “severe mental disorder”, which could guide professionals in relation to future interventions.

With regard to methodology, most studies do not record sociodemographic information pertaining to the authors of the blogs/vlogs. This is due to the difficulties in establishing direct contact with the authors, which would require a significant investment of time, the potential loss of participating blogs/vlogs due to a lack of response and the need to obtain approval from an ethics committee, which is usually not needed for public blogs/vlogs studies. As a result, it is challenging to determine the characteristics of the authors under investigation. The profiles of bloggers/vloggers are unknown, making it very difficult to analyse the intersection of having a mental disorder with gender, gender identity, sexual orientation, socioeconomic status, membership in minority groups, country of origin and other variables of interest in blogs/vlogs [61, 62]. Thus, only one study has been conducted on vlogs created by African American individuals with depression [41]. Furthermore, the lack of contact with content creators limits the scope of studies to the image they present in their blogs/vlogs, without insight into the personal perspectives of the authors themselves. It is plausible that the motivations, mental health status, benefits and challenges of the authors diverge from their online expressions. In fact, very few articles explored the negative aspects of blogs/vlogs. It is unlikely for bloggers/vloggers to express the harmful personal effects of blogs in their online presentation of themselves. The lack of, for instance, in-depth interviews or standardised scales with the authors themselves represents a significant gap in this field.

Moreover, most articles have employed purposive sampling or systematic searches. The difficulty in defining and obtaining a representative sample is inherent in the nature of the present object of study. However, to obtain a representative sample, we advise to select certain platforms as the object of study, perform a systematic search (as is done in systematic reviews), use selection criteria and employ a randomized final selection if the number of pieces of content is too high for the analysis to be performed. This method has been the most fruitful approach in the reviewed articles and has been successfully employed by Coll-Florit et al. [48].

Furthermore, a considerable variety of blog/vlog research methods is observed, including automatic analysis through machine learning, qualitative analysis of narratives, metaphors or words analysis, or combinations thereof [36, 38, 39, 48]. This is a strength, as it allows for the analysis of blogs/vlogs and the extraction of diverse and enriching information. However, it also presents challenges in terms of integrating and comparing the results. In addition to the development of novel methodologies, it would be beneficial to develop methodologies that can be applied to various types of blogs/vlogs on different platforms to facilitate comparison. Potential approaches include the creation of qualitative analysis templates, the analysis of specific indicators through questionnaires completed by the researcher or the implementation of established automatic analysis procedures that can be replicated. Furthermore, the combination of automatic analysis for the selection of relevant pieces of information with manual qualitative analysis may prove to be a fruitful avenue for future research, as illustrated by Van de Ven and Van-Nuenen [36].

## Limitations

The limitations of this systematic review are largely contingent on the previously mentioned research gaps. The results presented in this study are limited to the image displayed by the authors on the Internet and there is a notable absence of intersectional analysis. The direct perspective has not been studied and, in many cases, even their country of origin is unknown. Similarly, there is a lack of research examining the comparative analysis of narrative characteristics across different platforms or the role of their affordances. Consequently, different blogs/vlogs have frequently been treated as if they were similar, however, evidence suggests otherwise [36].

Furthermore, grey literature was not under analysis and articles in languages other than English were not considered, potentially introducing language bias into the analysis.

## Future Lines of Action

First, to gain insight into the narratives developed in these media and their creators, it would be beneficial to explore the opinions of the authors through interviews or the development of methodologies that examine data not shared in online media. This type of research would provide valuable information on mental health bloggers and vloggers.

We may also consider that the critique of health services extracted from the content and the benefits related to their use paves the way for a novel approach to intervention by health professionals. By engaging with this type of media, we can gain invaluable insight into the needs of individuals with SMI and CMI, enabling us to tailor our professional practice to meet their specific requirements. Of the reviewed studies, only one [34] implements an intervention using blogs, underscoring the necessity of this approach as a therapeutic measure. In this sense, intervention methods could be developed based on the use of these platforms, such as group discussions of public blogs/vlogs. For example, discussion groups could address topics that have previously been explored by other content creators, or they could encourage users to develop their own blogs or vlogs. The latter would facilitate personal development while also providing a platform with assist and connect to others.

It is also worthwhile to consider not only the practice of these forms of creative activity but also their impact on consumers. In this regard, it would be beneficial to investigate the advantages and disadvantages associated with the consumption of such media or how they influence the recovery and well-being process of consumers. In light of the rapid advancement of technologies in our context, they could potentially serve as a valuable medium for both patients and health professionals.

## Electronic Supplementary Material

Below is the link to the electronic supplementary material.

Supplementary Material 1. Characteristics and methodological quality of the study sample.

## Data Availability

Data will be made available upon request.
