# Supplementary material for: A Systematic Review of the Use of Blogs and Vlogs as Self-Expressors for People with Serious and Common Mental Illness
Source: Psychiatr Q. 2025 May 10;97(3):599–619. doi: 10.1007/s11126-025-10158-2 (PMC13518503; doi:10.1007/s11126-025-10158-2)
Supplement: Supplementary file 1 [file 11126_2025_10158_MOESM1_ESM.pdf]

Electronic Supplementary Material.

Characteristics and methodological quality of the study sample

Author	Sample (Country where the study was carried out, Description of the sample (N) and Sampling)	Descriptive Sample Data Participants: Diagnosis/groups, Gender, Age, Socioeconomic Status, Employment Status. Blogs: Website or Platform, Number of posts, Words (Corpus), Comments. Vlogs: Website or Platform, Mean Length, Mean Views, Comments.	Aims of the Study	Methodology: Type of Study and Data Analysis	Journal	MMAT (Only those having passed the two screening questions will be assessed.)
Coll-Florit et al. (2021a)	- Spain.<br>- Blogs written by patients with severe mental disorder, produced from June 2007 to March 2018 (N=73).<br>- Blogs: Purposive sampling, systematic search and virtual snowballing. Corpus: simple random sampling.	Participants:<br>- Bipolar disorder (34,2 %), schizophrenia (30,1 %), major depression (28,8 %) and obsessive compulsive disorder (6,9 %).<br>- No gender information.<br>- No age information.<br>- No socioeconomic status information.<br>- No employment status information.<br><br>Blogs:<br>- No website or platform information.<br>- 3,539 posts from patients and professionals. No	To identify and analyse the predominant metaphors used in online blog discourse to talk about mental illness and related experiences, and those used to conceptualize health professional practice, including comparative analysis between patients and professionals.	- Mixed study.<br>- Qualitative: in-depth analysis, hypothesising metaphors and metaphor analysis (Coll-Florit & Climent, 2019).<br>- Quantitative: ANOVA for differences between patient subgroups.	Culture, Language and Representation	5

		information on the number of publications written by patients only.<br>- 745 514-word corpus.<br>- No comment information.				
Coll-Florit et al., (2021b) (metaphors of depression)	- Spain.<br>- Blogs describing the authors' personal experiences with depression, written in Catalan between 2016 and 2018 (N=23).<br>- Purposive sampling.	Blogs:<br>- 12 different websites.<br>- No information on the number of publications.<br>- 13,641-word corpus.<br>- No comment information.	To analyze whether the metaphors of depression detected in previous studies and in other discursive genres are also used in a corpus of first-person testimony blogs written in Catalan, and in what proportions	- Qualitative study.<br>- Manual metaphor analysis as described in Coll-Florit et al., (2019).	Metaphor and Symbol	5
Coll-Florit, M. & Climent, S. (2022)	- Spain.<br>- Blogs written by patients with severe mental disorder, produced from June 2007 to March 2018 (N=73).<br>- Blogs: Purposive sampling, systematic search and virtual snowballing. Corpus: simple random sampling.	Participants:<br>- Bipolar disorder (34,2 %), schizophrenia (30,1 %), major depression (28,8 %) and obsessive compulsive disorder (6,9 %).<br>- No gender information.<br>- No age information.<br>- No socioeconomic status information.<br>- No employment status information.<br><br>Blogs:<br>- No website or platform information.<br>- No information on the number of publications.	To analyse how metaphors of war and journey are used in online discourses about mental health in Spanish by patients and profesionals	- Mixed study.<br>- Qualitative: in-depth analysis of metaphors about severe mental disorders and subsequent division into categories.<br>- Quantitative: Means contrast. ANOVA for intra-group differences.	Metaphor and the Social World	5

		- 745 514-word corpus.<br>- No comment information.				
Conneely et al. (2020)	- USA and Canada.<br>- The first 10 blogs, with more than 20 posts, that appeared in Google searches using the terms "depression blog", carried out between May and September 2016 (N=10).<br>- Purposive sampling.	Participants:<br>- 100% depression (from 1 year to "decades").<br>- Women (70%) and men (30%).<br>- Age range from the 20s to the 50s.<br>- No socioeconomic status information.<br>- Employed (70%), rest unspecified.<br>Blogs:<br>- No platform information.<br>- From 31 to over 100 posts.<br>- No corpus information.<br>- No comment information.	To analyze the medical and the moral view of depression in blogs	- Qualitative study.<br>- Thematic analysis.	Social Theory and Health	5
Egher (2019)	- No information.<br>- Entries (n=25) of bipolar disorder platforms (n=2) which covered the treatment of BD, broadly understood, and which had more than 30 comments each. US platforms: 1st from a medical professional and caregiver, 2nd from a person diagnosed with BD (referral from the BD community).<br>- Purposive sampling.	Participants:<br>- 100% Bipolar disorder.<br>- No gender information.<br>- No age information.<br>- No socioeconomic status information.<br>- No employment status information.<br>Blogs:<br>- US platforms: 1st from a medical professional and caregiver, 2nd from a person diagnosed with BD (referral from the BD	To find out how bloggers positioned themselves in relation to medical perspectives on treatment, how they attempted to develop alternative narratives, and how they negotiated the development of new assumptions about treatment efficacy.	- Qualitative study.<br>- Computer-mediated discourse analysis (Herring, 2012) and sociolinguistics (Blommaert, 2005).	Health	3

		community).<br>- No information on the number of publications.<br>- No corpus information.<br>- More than 30 comments each.				
Egher (2020)	- EEUU and UK.<br>- Bloggers diagnosed with bipolar disorder who appear in the first page of google searches, are highly successfull, and has become entrepreneurial selves (N=3).<br>- Purposive sampling.	Participants:<br>- 100% bipolar disorder (two decades since diagnosis).<br>- 100% women.<br>- No age information.<br>- No socioeconomic status information.<br>- No employment status information.	To expand the notion of interactional expertise in blogs by focusing on its bi-directional character and by considering the effects of the medium through which it is articulated	- Qualitative study.<br>- Thematic analysis.	Science & Technology Studies	5
Fineberg et al. (2016)	- No information.<br>- The most recent 150+ word entry from blogs in English of people who self-identified as having schizophrenia or schizoaffective disorder (n=54), blogs from other illness (n=219) and non-illness groups (n=141).<br>- Purposive sampling.	Participants:<br>- Schizophrenia or schizoaffective disorder: Psychosis (100%). Other illness: Morgellon's (16,9%), Depression (21,9%), Aspie (20,6%), Spinal Cord Injury (18,7%), Cancer (21,9%). Non-illness groups: Evangelical (34%), Anti-religion (34,8%) and Conspiracy (31,2%).<br>- Psychosis group: Women (59.3%) and men (40.7%).<br>- No age information.<br>- No socioeconomic status	To investigate the relationship between lexical features and specific aspects of psychosis.	- Quantitative study.<br>- Frequency counts of word categories, ANOVA, correlations, and principal component analysis.	Psychological Medicine	3

		information.<br>- No employment status information.				
Johnson et al. (2021)	- No information.<br>- YouTube videos Black/African American participants who shared their experiences with living with depression or depression symptomatology (N=50).<br>- Purposive sampling.	Participants:<br>- 100% depression.<br>- Gender: 42% men; 58% women.<br>- Age range: teenage years to mid-30's.<br>- Diverse socioeconomic status.<br>- No employment status information.<br><br>Videos:<br>-Youtube.<br>- 13 min mean length (range: 1.75 min to 67.2 min).<br>- 894,949 total views.<br>- No comment information.	To understand what are the disease management experiences of BYA who identify as having clinical depression or depression symptomatology on YouTube.	- Qualitative study.<br>- Thematic analysis.	Informatics for Health and Social Care	5
Kotliar (2015)	- No information.<br>- Blogs written by women who explicitly self-identified as having depression (N=5).<br>- Purposive sampling, systematic search and virtual snowballing.	Participants:<br>- 100% depression.<br>- 100% women.<br>- No age information.<br>- No socioeconomic status information.<br>- No employment status information.<br>Blogs:<br>- No website or platform information.<br>- 350 mean posts per blog	To examine the ways in which people represent, interpret and explain their depression on the Internet, and to portray the unique and often paradoxical links between the reclusiveness that characterises depression and extreme exposure on	- Qualitative study.<br>- Thematic analysis (Braun & Clarke, 2006; Vaismoradi, Turunen, & Bondas, 2013) with an interpretive-constructivist approach (Heikkinen, Huttunen, & Kakkori, 2000; Robinson, 2001).	Qualitative Health Research	5

		over a 2–3-year period.<br>- No corpus information.<br>- No comment information.	blogs written by women with major depression.			
MacLean et al. (2017)	- No information.<br>- YouTube videos uploaded by lay people in English with at least 50,000 views, found by using the search term "generalized anxiety disorder" in October 2016 (N=62).<br>- Systematic search.	Participants:<br>- 100% "generalized anxiety disorder".<br>- No gender information.<br>- No age information.<br>- No socioeconomic status information.<br>- No employment status information.<br>Vlogs:<br>- Youtube.<br>- No length information.<br>- 352,365 mean views.<br>- No comment information.	To describe the content of the most popular YouTube™ videos on generalized anxiety disorder uploaded by both lay people and professionals, focusing on the symptoms, treatments and causes of anxiety mentioned.	- Mixed study.<br>- Qualitative: Thematic analysis.<br>- Quantitative: Chi Square and Fisher's exact test.	International Journal of Social Psychiatry	5
Mandiberg & Gates (2017)	- USA.<br>- People with SMI (peers), hired as peer workers by six mental health agencies (N=16).<br>- Purposive sampling.	Participants:<br>- No diagnosis information.<br>- No gender information.<br>- No age information.<br>- No socioeconomic status information.<br>- No employment status information.<br>Blogs:<br>- Own platform.<br>- 6.7 mean posts (ranging from 26 to 0).<br>- No corpus information.<br>- No comment information.	To develop and analyse the uses of a asynchronous interactive website with blog functionalities to facilitate a sense of community and mutual support among workers with severe mental illness from disparate locations.	- Qualitative study. Naturalistic observation (Erlandson, Harris, Skipper and Allen, 1993).<br>- Descriptive analysis.	American Journal of Psychiatric Rehabilitation	1

Mandla et al. (2017)	- USA (n=10), South Africa (n=1) . Rest unknown. (UK platforms).<br>- Blogs (n=45) from people identified as individuals with bipolar disorder (n=22) who provided sufficient information about problems and symptoms, diagnosis experience, causes and experiences/opinions of treatment.<br>- Purposive sampling, systematic search and virtual snowballing.	Participants:<br>- No diagnosis information.<br>- Women (68,2 %), men (18,2 %) and unspecified (13,6 %)<br>- Age range: 35-50 (22,7%), over 50 (31,8%), rest unknown (54,5%).<br>- No socioeconomic status information.<br>- No employment status information.<br>Blogs:<br>- 12 different websites.<br>- No information on the number of publications.<br>- No corpus information.<br>- No comment information.	To explore how BD is presented on the Internet by people who identify themselves as having the condition.	- Qualitative study.<br>- Thematic analysis.	Issues in Mental Health Nursing	5
Naslund et al. (2014)	- No information.<br>- Comments (n=3,044) posted to videos uploaded by individuals (n=19) who self-identified as having schizophrenia, schizoaffective disorder, or bipolar disorder.<br>- Purposive sampling, systematic search.	Participants:<br>- Bipolar Disorder (42,1%) , Schizoaffective Disorder (36,8%) and Schizophrenia (21,1%).<br>- Gender: Women (36,9%), men (21,1%), rest unspecified. Divided into groups: Bipolar Disorder group (women, 5,3 %; men, 5,3 %; rest unspecified), Schizoaffective Disorder group (women, 21,1%; men, 5,3%; rest unspecified), Schizophrenia	To explore the phenomenon of individuals with severe mental illness uploading videos to YouTube and posting and responding to comments as a form of naturally occurring peer support.	- Qualitative study. Online ethnography.<br>- Thematic analysis.	PLoS ONE	5

		group (women, 10,5%; men, 10,5%).<br>- Age range: 18–35 years (78,9%), 36–55 years (21,1%).<br>- No socioeconomic status information.<br>- No employment status information.<br>Vlogs:<br>- Youtube.<br>- No length information.<br>- 19786 mean views (range of 5,433 to 88,654).<br>- 3,044 coments (range of 27 to 592).				
Romakkaniemi & Kilpeläinen (2015)	- Finland.<br>- Blog posts from 2 blogs authored by people with depression, written in Finnish during one year period (2011), (N=2).<br>- Purposive sampling.	Participants:<br>- 100% Depression.<br>- 100% women.<br>- No age information.<br>- No socioeconomic status information.<br>- No employment status information.<br><br>Blogs:<br>- Total pages: 318	To find out meaningful elements in the encounters between profesionals and depressed people that promote or are harmful for recovery from major depression	- Qualitative study.<br>- Thematic analysis.	Social Work in Mental Health	5
Sangeorzan et al. (2019)	- No information.<br>- Videos (n=30) uploaded by individuals (n=28) with self-identified SMIs discussing their experience of vlogging.<br>- Purposive sampling,	Participants:<br>- Bipolar disorder (42,8%), schizophrenia (28,6%), schizoaffective disorder (7,1%) and major depression (7,1%). Mixed	To examine the impact of vlogging on individuals with an SMI who upload mental health videos.	- Qualitative study.<br>- Interpretative Phenomenological Analysis (IPA) (Smith et al., 1999).	Journal of Affective Disorders	4

	systematic search and virtual snowballing.	diagnoses: bipolar disorder and major depression (3,6%), bipolar disorder and schizoaffective disorder (3,6 %), schizophrenia and major depression (3,6%) and schizophrenia and schizoaffective disorder (3,6 %).<br>- No gender information.<br>- No age information.<br>- No socioeconomic status information.<br>- No employment status information.<br>Vlogs:<br>- Youtube.<br>- 10 min mean length.<br>- No views information.<br>- No comment information.				
Van de Ven, I., & Van-Nuenen, T (2022)	- No information. Although there are UK and international platforms.<br>- Blogs tagged as "depression" in 3 platforms: time-to-change.org (n=601 posts), Sane.org.uk (n=1490 posts) and the r/depression subreddit (n=706231 posts) from 2007 to 2019.<br>- Purposive sampling, systematic search.	Participants:<br>- 100% depression.<br>- No gender information.<br>- No age information.<br>- No socioeconomic status information.<br>- No employment status information.<br>Blogs:<br>- 3 platforms: TTC (entries tagged with the tag "depression"), SANE (peer to peer blogs) and Reddit	To analyze the depression discourses online of lay people.	- Mixed study.<br>- Digital hermeneutics approach. Scaled readings on five scales: 1. Platform Hermeneutics, 2. Contextual reading (Term-Frequency - Inverse Document Frequency (TF-IDF), 3. Distant reading (Topic Modelling), 4. Hyper-Reading (Concordance views), 5. Close Reading (close reading of a "telling case"). A mixture of	Medical Humanities	4

		(subreddit "r/depression").<br>- TTC (601 posts), SANE (1490 posts) and Reddit (706231 posts).<br>- TTC (492929-word corpus), SANE (581535-word corpus), Reddit (191249252-word corpus).<br>- Reddit (3251544 comments), no information on the comments in TTC and SANE platforms.		qualitative and machine learning analyses.		
Woloshyn & Savage (2020)	- No information (UK, Europe, Australia and North America but not formally analysed).<br>-YouTube videos of individuals who self-identified with the diagnosis of borderline personality disorder (N=349).<br>- Purposive sampling, systematic search.	Participants:<br>- 100% Bordeline Personality Disorder.<br>- No gender information.<br>- No age information details, though it mentioned that they were young adults.<br>- No socioeconomic status information.<br>- No employment status information.<br>Vídeos:<br>- Youtube.<br>- 11.1 min mean length.<br>- 10791.1 mean views.<br>- 43.3 mean comments.	To describe the predominant messages in YouTube videos of individuals who self-identify with a diagnosis of BPD or EUPD.<br>To examine the characteristics of the vloggers and their videos.<br>To find out the motives, contexts and life experiences cited by the vloggers in their videos.	- Mixed study.<br>- Qualitative: content analysis.<br>- Quantitative: descriptive statistics for continuous video posting functions, with dichotomous coding for motivation and video content. Spearman's Rho for relationships between pairs of continuous and discrete variables.	Digital Health	4
Xu, K. & Li, X. (2023)	- China.<br>- Stories posted from December 2020 to September 2021 in the "Philosophical Treatment of Depression"	Participants:<br>- 100% Depression.<br>- No gender information.<br>- No age information.<br>- No socioeconomic status	To study the largest online depression community in China in order to understand how people with depression	- Qualitative study.<br>- Systematic thematic narrative analysis.	Qualitative Health Research	5

group of Douban platform (N=160).<br>- Simple Random Sampling.	information.<br>- No employment status information.<br>Blogs:<br>- Douban.<br>- 160 posts.<br>- No corpus information detailed, but more than 400 words per post.<br>- No comment information.	narrate their experiences and make sense of the causes of their trauma in the Chinese social context.
